# Comprehensive Assessment and Trading Mechanism of Carbon Sink in China’s Marine Aquaculture

**DOI:** 10.3390/biology14060648

**Published:** 2025-06-03

**Authors:** Xuan Yu, Haonan Guo, Qi Chen

**Affiliations:** 1 Business School, Ningbo University, Ningbo 315211, China; ghaonan2024@163.com (H.G.); chenqi2@nbu.edu.cn (Q.C.); 2 Donghai Academy, Ningbo University, Ningbo 315211, China

**Keywords:** carbon sink, marine aquaculture, comprehensive assessment, trading mechanism

## Abstract

This study develops species-specific assessment models for carbon sink in marine aquaculture (CSMA) using provincial data from China’s coastal regions (2004–2023). Key findings are as follows: (1) Cumulative CSMA reached 46.3618 million tonnes, exhibiting three growth phases—initial fluctuations (2004–2008), rapid growth (2008–2015), and optimization and maturation (2015–2023). (2) Species contributions were heterogeneous: shellfish dominated at 45%, followed by shrimp (24%), fish (15%), crab (11%), and algae (5%). (3) Spatially, Guangdong, Fujian, and Shandong consistently lead in CSMA; Guangxi, Liaoning, and Zhejiang form a second tier, whereas Hebei, Hainan, and Jiangsu remain at the lower end. (4) Province-specific dominance patterns were observed: shellfish-dominant pattern in Shandong, Fujian, and Liaoning; shrimp-dominant pattern in Hebei and Hainan; shellfish-and-shrimp dual-cores in Guangdong and Guangxi; and a multifaceted profile in Jiangsu and Zhejiang. To facilitate the realization of CSMA’s value, we propose a dedicated trading mechanism. Based on the derivations from the effect analysis model and the illustrative case studies, we explore the potential economic and ecological benefits of CSMA trading.

## Simple Summary

This study assesses China’s marine aquaculture as a carbon sink over the period 2004–2023. By analyzing five key cultured groups—shellfish, shrimp, fish, crabs, and algae—we quantified regional carbon sinks and delineated each group’s contribution. Results show that total carbon sink capacity more than doubled during this period, with shellfish alone contributing almost half (45%) of the cumulative sequestration. Different provinces exhibited distinct trends, some relying primarily on shellfish farming, others on shrimp or mixed aquaculture systems. To translate these ecological gains into practice, we propose a dedicated carbon sink trading mechanism. Our analysis indicates that this mechanism could provide additional income to aquaculture operators and incentivize sustainable practices, thereby enhancing marine habitat quality and advancing China’s climate objectives.

## 1. Introduction

The ocean, Earth’s largest active carbon reservoir, is pivotal in regulating the global climate [1,2,3]. Empirical research shows that the ocean stores about 93% of global CO_2_ and sequesters roughly 30% of human-generated CO_2_ emissions each year [4]. With terrestrial carbon sinks becoming increasingly unstable under climate change, meeting global mitigation targets demands greater attention to the carbon-sequestration functions of nearshore marine ecosystems [5,6,7].

The marine carbon sink—commonly called “blue carbon” to contrast with terrestrial “green carbon”—encompasses the processes, activities, and mechanisms by which marine life and ecosystems draw down atmospheric CO_2_ and store it in the ocean’s depths [8,9,10]. Compared with green carbon, blue carbon can sequester larger quantities of CO_2_, cycle it over longer timescales, and retain it more permanently [11,12,13]. Blue carbon is primarily driven by three “ocean pumps”. The biological pump describes how marine life—phytoplankton, photosynthetic algae, zooplankton, and microbes—transforms atmospheric CO_2_ into particulate organic carbon that sinks from the surface to the deep-sea floor, where it is broken down and sequestered [14,15,16]. The solubility pump uses physical processes—turbulent mixing, gas diffusion, and heat exchange—to dissolve CO_2_ into surface waters and transport it to the deep ocean, particularly in cold, high-salinity zones where dense water formation stores carbon on millennial timescales [17,18]. Finally, the carbonate pump occurs when marine microorganisms precipitate calcium carbonate during growth and metabolism, forming mineral layers—such as laminated seafloor carbonates and cold-seep crusts—that permanently sequester inorganic carbon in sediments [19,20,21].

The carbon sink of marine aquaculture (CSMA) encompasses processes by which farming activities boost CO_2_ uptake or utilization by aquatic organisms and then permanently remove that fixed carbon from the water column through harvest [22,23]. For example, shellfish capture carbon in two main ways: by biomineralizing calcium carbonate in their shells and by accumulating organic carbon in their tissues, with shell deposition serving as the primary long-term inorganic carbon sink because buried shells resist dissolution [24,25]. Algae capture CO_2_ through photosynthesis, transforming dissolved inorganic carbon into organic biomass while releasing dissolved organic carbon [26,27]. Fish and shrimp lock away carbon largely in their growing tissues and skeletons [28], whereas crabs sequester carbon chiefly by depositing calcium carbonate during each molting cycle [29]. Although the mechanisms differ among species, they all function as part of the broader biological pump. From a holistic carbon-cycle perspective, marine aquaculture does incur carbon emissions—from feed production and energy use to infrastructure investment and vessel operations [30,31]. However, many studies also have shown that marine aquaculture significantly enhances coastal carbon cycling and helps offset human-driven CO_2_ emissions [32,33,34,35].

As the world’s leading producer of farmed marine species, China’s aquaculture output reached 23.956 Mt (million tonnes) in 2023, representing more than 70% of global production. Against the backdrop of China’s “dual-carbon” targets—peaking CO_2_ emissions by 2030 and achieving carbon neutrality by 2060—marine aquaculture stands out as a promising mitigation strategy, though comprehensive life-cycle emissions (e.g., from feed production and energy use) and other trade-offs must be considered alongside its benefits. In this study, we use provincial-level data from China’s coastal regions (2004–2023) to estimate total CSMA, describe its temporal evolution, and examine spatial and species-specific differences. To unlock CSMA’s ecological and economic potential, we propose a dedicated trading mechanism and employ an effect analysis to quantify its economic, environmental, and synergistic benefits.

## 2. Materials and Methods

### 2.1. Study Area

**Figure 1:**
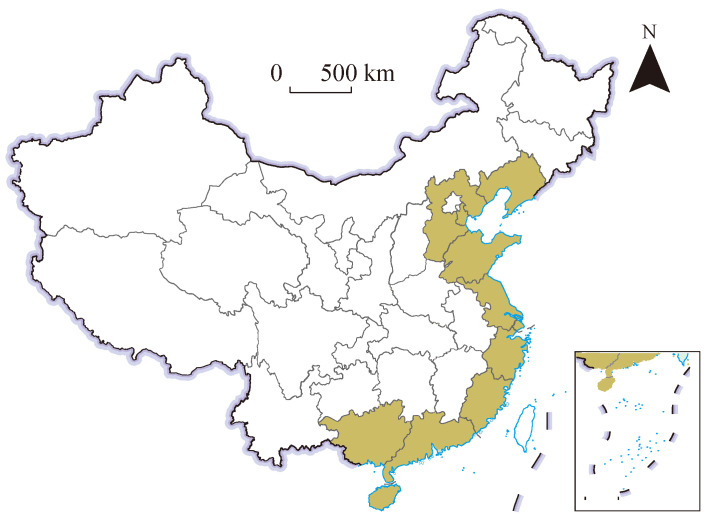
Study area.

This study covers China’s coastal zone (Figure 1), including eight provinces (Liaoning, Hebei, Shandong, Jiangsu, Zhejiang, Fujian, Guangdong, and Hainan), one autonomous region (Guangxi), and two municipalities (Tianjin and Shanghai). Hong Kong, Macao, and Taiwan were excluded owing to data unavailability. Although Shanghai and Tianjin hold provincial status administratively, their aquaculture outputs are minimal compared to the other nine regions; therefore, they were omitted from the regional CSMA analysis.

### 2.2. Estimation of Total CSMA

We focused on five key aquaculture categories—fish (8.6%), shrimp (7.4%), crab (1.2%), shellfish (68.7%), and algae (12.0%)—which together accounted for approximately 97.9% of China’s total marine aquaculture output in 2023. To examine temporal trends, we compiled marine aquaculture data for China’s coastal provinces from 2004 to 2025, drawing on the EPS database (https://www.epsnet.com.cn/, accessed 10 March 2025). CSMA for shellfish and algae was estimated in accordance with the Ministry of Natural Resources guidelines (HY/T 0305-2021) [36]. Under these protocols, shellfish CSMA includes carbon sequestered in both shells and soft tissues, with their respective proportions remaining largely constant across varied farming conditions [24]. Accordingly, the CSMA of shellfish species *k* is computed using the following equation:

(1) $$S_{k}=Y_{k}R_{d}\left(M_{rt}cM_{rt}+M_{s}cM_{s}\right)$$

In Equation (1), *S_k_* denotes the CSMA of shellfish species *k*; *Y_k_* is the yield of shellfish species *k*; *R_d_* is the wet-to-dry weight conversion factor; *M_rt_* is the proportion of soft tissue (dry weight basis); *cM_rt_* is the carbon content of soft tissue; *M_s_* is the proportion of shell (dry weight basis); and *cM_s_* is the carbon content of shell. Wet-to-dry weight conversion factors, tissue/shell mass ratios, and carbon contents for common bivalve species are listed in Table 1.

Algal CSMA is determined exclusively by the carbon content of algal biomass produced through photosynthetic fixation of dissolved inorganic carbon [37]. For each algal species *i*, CSMA is calculated as follows:

(2) $$S_{i}=Y_{i}R_{s}W_{ci}$$

In Equation (2), *S_i_* represents the CSMA of the algae species *i*; *Y_i_* is the yield of that species; *R_s_* is the wet-to-dry weight conversion coefficient for algae; and *W_ci_* is the carbon content fraction of the algae. Table 2 summarizes these wet-to-dry conversion factors and carbon contents for the major cultivated algae.

CSMA for fish, shrimp, and crab was quantified according to the model of Jiao et al., as follows:

(3) $$S_{j}=Y_{j}Z_{cj}$$

where *S_j_* is the CSMA of group *j* (fish, shrimp, or crab); *Y_j_* is the corresponding aquaculture yield; and *Z_cj_* is the carbon sink coefficient. The species-specific sequestration coefficient *Z_cj_* is valued at 0.23 for fish, 0.43 for shrimp, and 1.26 for crab [38].

After estimating the total CSMA, we further calculate the economic value of CSMA. Using the shadow price better reflects the true economic contribution of marine carbon sinks, while the market price is more conservative and practical for estimating near-term trading revenue. Therefore, based on Chai et al.’s calculations of carbon sink prices in China’s marine fisheries for 1979–2022 and aligned with our sample period, we adopt the average annual price for 2004–2022—CNY 288.72 per t CO_2_—as the carbon price for estimating CSMA economic value [39].

### 2.3. Effect Analysis Model

We employ an effect analysis model to quantify the economic and ecological impacts of CSMA trading. We consider a tripartite economic framework involving coastal governments, marine aquaculture firms, and external CSMA buyers. The model incorporates the following core functions and variables:

- Production Function: Q=f(L,K,E). The output of marine aquaculture (*Q*) is modeled as a function of labor input (*L*), capital input (*K*), and marine ecological quality (*E*), where enhancements in *E* are assumed to boost *Q*.
- CSMA Capacity Function: C=g(Q,E). The amount of CSMA (*C*) is defined as an increasing function of production output (*Q*) and ecological quality (*E*), reflecting that higher *Q* and improved *E* enhance carbon sink capacity.
- Environmental Governance Cost Function: M=h(E). The cost (*M*) incurred by coastal authorities or aquaculture firms to improve ecological quality (*E*) is modeled as an increasing function of *E*. This cost function satisfies $h^{\prime }>0$, $h^{\prime \prime }>0$; that is, improving *E* requires additional investment, with increasing marginal costs.
- Carbon Price: $p_{c}$. It is treated as an exogenous variable, set by market supply and demand conditions.

## 3. Results

### 3.1. Total Variation of CSMA

Over the 2004–2023 interval, China’s total CSMA reached 46.3618 Mt. At an average carbon price of CNY 288.72 per t CO_2_, the total economic value of these CSMAs is approximately CNY 13.386 billion.

**Figure 2:**
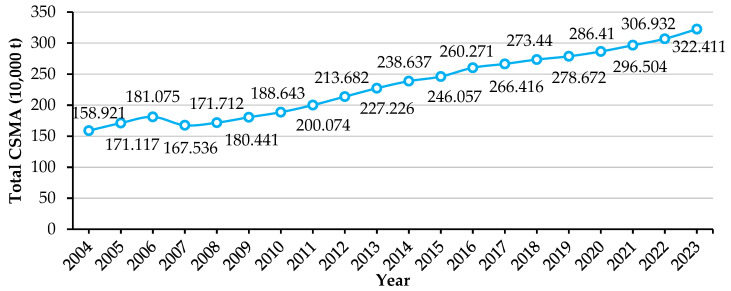
Total CSMA variation in China, 2004–2023.

Figure 2 shows that CSMA increased from 1.58921 Mt in 2004 to 3.22411 Mt in 2023, representing a cumulative growth of 102.9%. The trend can be divided into three phases. Between 2004 and 2007, CSMA fluctuated between 1.58 and 1.81 Mt, including a notable 7.5% drop in 2007. This decline was primarily caused by extensive Ulva blooms and red tide events, which smothered shellfish beds and disrupted aquaculture operations. From 2008 to 2015, CSMA entered a rapid growth phase, with annual increases averaging 5.3% and a rise to 2.46057 Mt in 2015—a 43.3% gain over 2008 levels. Between 2015 and 2023, CSMA entered an optimization and maturation phase. Although annual growth slowed to 3.4%, total CSMA nonetheless reached new highs each year.

### 3.2. Differences in CSMA Capability

#### 3.2.1. Species-Level Differences

**Figure 3:**
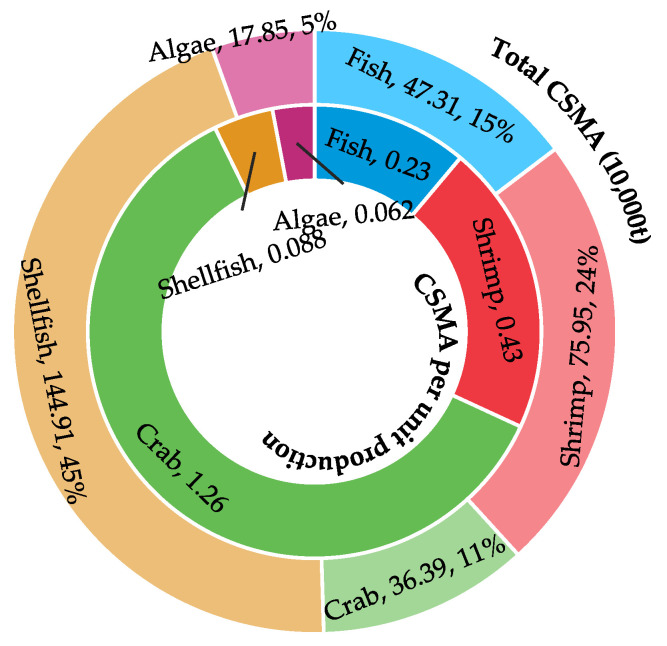
Species-specific differences in total CSMA and carbon sink coefficients in China, 2023.

Figure 3 displays the 2023 distribution of CSMA across different species. Significant heterogeneity exists among the five major cultured groups, as their carbon sink coefficients (CSMA per unit production) diverge from their total CSMA contributions. Crabs demonstrate the highest sequestration efficiency (1.26 t CSMA per t production) but contribute merely 0.3639 Mt (11%) to total CSMA. In contrast, shellfish—despite having the lowest coefficient (0.088 t CSMA per t)—contribute 1.4491 Mt (45%) to total CSMA, establishing them as the dominant source by scale. Shrimp occupy an intermediate position, characterized by a coefficient of 0.43 t CSMA per t and a total CSMA of 0.7595 Mt (24%), thereby balancing efficiency and scale.

#### 3.2.2. Regional Differences

**Figure 4:**
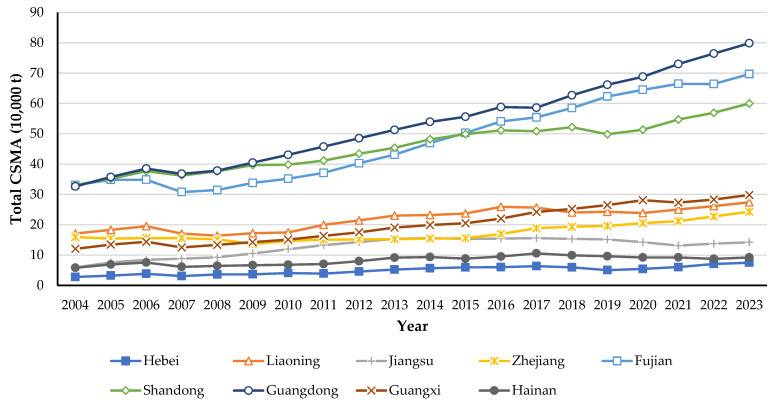
Provincial variation of total CSMA, 2004–2023.

Figure 4 summarizes the provincial CSMA totals from 2004 to 2023. A distinct “stepwise differentiation” pattern is evident. In 2023, Guangdong (0.7983 Mt), Shandong (0.5999 Mt), and Fujian (0.6969 Mt) collectively accounted for 65% of national CSMA, constituting the leading tier. Liaoning, Zhejiang, and Guangxi made up the second tier with a combined share of 25.3%, whereas Hebei, Hainan, and Jiangsu formed the lowest tier, contributing only 9.6%. Growth rates further underscore the regional disparities in CSMA development. Among the nine coastal provinces, Hebei exhibited the highest average annual growth at 5.92%, followed by Guangxi (5.02%), Jiangsu (5.03%), and Guangdong (4.86%), all exceeding the national average of 4.01%. Fujian’s growth (4.11%) was also slightly above average. In contrast, Shandong (3.23%), Hainan (2.84%), Liaoning (2.69%), and Zhejiang (2.36%) lagged behind the national mean.

At the regional scale, total CSMA exhibits the following two prominent spatiotemporal evolution patterns: growth-pole restructuring and heterogeneous volatility. Regarding growth poles, Guangdong surpassed Shandong in 2005 to become the leading CSMA province, and Fujian overtook Shandong in 2015 to become the second-largest contributor to national CSMA. In terms of volatility, Fujian exhibited the highest interannual fluctuation rate; in 2007, natural disturbances—primarily typhoons and harmful algal blooms—severely impacted its aquaculture sector, resulting in an 11.7% decline in CSMA. In contrast, Zhejiang’s CSMA annual growth stabilized at approximately 4.9% between 2015 and 2020, primarily due to effective coastal eutrophication management. By reducing nutrient inputs and mitigating the occurrence of harmful algal blooms, water quality in coastal areas improved, which created more favorable conditions for aquaculture. Improved environmental quality can enhance survival rates and productivity of cultured species, thereby contributing to more stable and sustained CSMA growth.

#### 3.2.3. Category–Region Interaction

**Figure 5:**
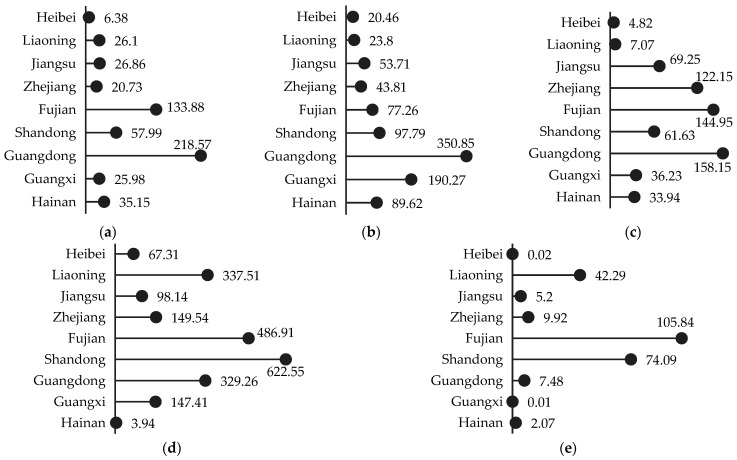
Category differences in total CSMA across nine coastal provinces of China, 2004–2023. (**a**) Fish; (**b**) shrimp; (**c**) crab; (**d**) shellfish; and (**e**) algae (unit: 10,000 t).

Figure 5 depicts the 2004–2023 CSMA totals categorized by species across nine coastal regions. At the national scale, shellfish farming dominates CSMA production, accounting for 22.7347 Mt (64.3% of the total). Shandong (6.2255 Mt), Fujian (4.8691 Mt), and Liaoning (3.3751 Mt) rank as the top three contributors. Fish and shrimp follow, contributing 5.5164 Mt (15.6%) and 9.4757 Mt (26.8%), respectively, while Guangdong stands out with fish (2.1857 Mt) and shrimp (3.5085 Mt). Crabs (6.2894 Mt, 17.8%) and algae (2.4692 Mt, 7.0%) exhibit regional clustering, with crab CSMA concentrated in Guangdong, Fujian, and Zhejiang (each >1.2 Mt) and algae CSMA predominantly in Fujian (1.0584 Mt).

Based on the patterns shown in Figure 5, the nine provinces can be classified into four empirical types according to their primary CSMA contributions: shellfish-dominant, shrimp-dominant, shellfish–shrimp dual-core, and comprehensive-multifaceted. Shellfish-dominant regions are exemplified by Shandong, Fujian, and Liaoning. Shrimp-dominant regions include Hebei and Hainan. The shellfish–shrimp dual-core region is exemplified by Guangdong and Guangxi, where shrimp and shellfish collectively define the carbon sink core through their absolute CSMA contributions. Finally, the comprehensive–multifaceted category encompasses Jiangsu and Zhejiang. Jiangsu exhibits a balanced distribution across crab, shrimp, and shellfish, reflecting coordinated multi-species development, whereas Zhejiang’s CSMA structure is dominated by crabs and shellfish, supplemented by fish and shrimp, resulting in a diversified sequestration profile.

### 3.3. CSMA Trading Mechanism

#### 3.3.1. Mechanism Design

(1) Pre-Transaction: Identification of Participants and Monitoring and Evaluation

**Figure 6:**
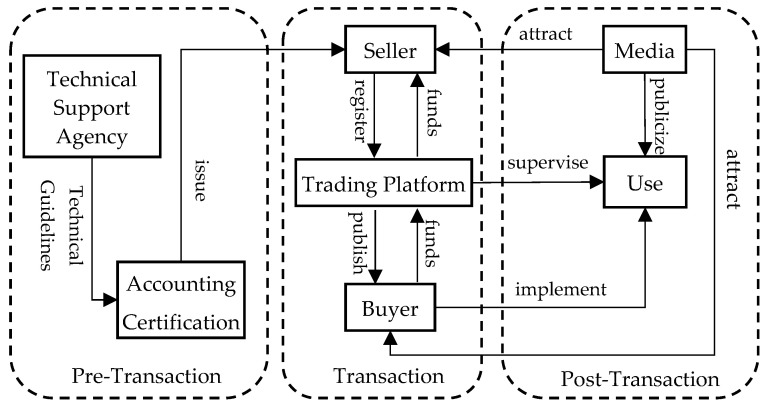
CSMA trading mechanism.

The CSMA trading process (Figure 6) comprises three phases: pre-transaction, transaction, and post-transaction. Two core questions must be addressed prior to trading: “Who trades?” (i.e., identification of market participants) and “What is traded?” (i.e., CSMA quantification and verification). During the pilot stage, supply is provided by government agencies that hold CSMA resources, while demand originates from voluntary buyers, such as enterprises or organizations seeking to offset emissions. Given the immaturity of early markets and the risk-averse nature of many firms, credible public bodies must serve as sellers to ensure legitimacy, while voluntary purchasers are driven by corporate social responsibility. As the market matures, marine aquaculture enterprises and individual farmers become CSMA suppliers, while entities become subject to mandatory emission constraints—such as power generation, chemical, construction material, and steel industries—and emerge as principal buyers. At full maturity, CSMA trading is integrated into China’s Certified Emission Reduction (CCER) mechanism and the Verified Carbon Standard (VCS), with governmental roles transitioning from active participation to regulatory oversight.

Effective monitoring and evaluation underpin the credibility of the market. We recommend leveraging integrated land–sea and ocean satellite systems to conduct comprehensive baseline surveys of key blue carbon habitats—such as mangroves, seagrass beds, and saltmarshes—and map their distribution and utilization. Subsequently, a CSMA quantification standard should be established, incorporating ecosystem types and characteristics. By employing visualization modeling, IoT sensors, and intelligent decision-support tools, a digital CSMA monitoring platform can track carbon stocks and fluxes in near real-time. Integration with broader “Digital Ocean” initiatives will facilitate cross-sectoral data sharing and enhance transparency among market participants.

(2) Transaction: Platform Development and Price Negotiation

The core of CSMA trading lies in the negotiation and execution of transactions on a dedicated platform. During the pilot stage, CSMA modules may be integrated into existing provincial public-resource trading platforms. In the mature phase, dedicated provincial CSMA exchanges and websites should be established under provincial government oversight, registered with the State-owned Assets Supervision and Administration Commission and the Ministry of Finance, and subsequently expanded to municipal levels. Platform responsibilities encompass setting trading rules, defining eligibility criteria, publishing listings, and ensuring compliance.

During price negotiation, sellers register CSMA valuation certificates on the platform, which subsequently publishes these offers publicly. Interested buyers engage with sellers through the platform, with pricing determined via bilateral agreements (single purchasers) or open auctions (multiple bidders). Upon finalization of terms, the platform facilitates payment settlement and title transfer.

(3) Post-Transaction: Supervision and Feedback Adjustment

The completion of a CSMA transaction does not conclude the regulatory process. Platforms and regulators must monitor buyers’ utilization of CSMA credits—such as corporate branding, “zero-carbon” product development, or public outreach—to prevent false advertising or unjustified cost transfers to consumers. Contractual performance must be enforced through mechanisms for breach remediation and intellectual property protection to mitigate third-party infringement.

A systematic feedback loop is imperative. Each transaction should undergo review to extract lessons, identify procedural weaknesses, and implement corrective measures for future trades. Media campaigns and stakeholder workshops can disseminate successful cases, attract new participants, and scale up the market. Through continuous supervision, adjustment, and outreach, the CSMA trading mechanism can transition from a pilot phase to a robust, transparent, and scalable blue carbon market.

#### 3.3.2. Effect Analysis

(1) Economic Effect

Without CSMA trading, the profit function of a marine aquaculture enterprise is defined as follows:

(4) $$\pi_{1}=p_{q}Q-wL-rK$$

In Equation (4), *Q*, *L*, and *K* denote marine aquaculture production, labor input, and capital input, respectively. *P_q_* denotes the price of marine aquaculture products, *w* represents the wage rate, and *r* indicates the rental rate of capital. When CSMA trading is implemented, marine aquaculture enterprises can sell the CSMA embedded in their products, modifying the profit function into the following expression:

(5) $$\pi_{2}=p_{q}Q+p_{c}C-wL-rK$$

A comparison of Equations (4) and (5) demonstrates that CSMA trading directly increases the profitability of marine aquaculture enterprises. Economically, the existence of a CSMA trading market incentivizes enterprises to expand production or enhance marine ecological quality to maximize *C*, thereby translating ecological value into economic gains.

(2) Ecological Effect

Based on the CSMA capacity function, the revenue from CSMA can be expressed as $R_{c}=p_{c}\cdot g(Q,E)$. The total differential of *R_c_* is as follows:

(6) $$dR_{c}=p_{c}\left(g_{Q}^{\prime }dQ+g_{E}^{\prime }dE\right)$$

Equation (6) indicates that CSMA revenue is jointly determined by the marginal contributions of *Q* (production output) and *E* (ecological quality). The optimal decision for economic agents involves selecting the environmental governance investment level *M* that maximizes net revenue:

(7) $$\max_{E}\;\pi =p_{q}Q+p_{c}g(Q,E)-wL-rK-h(E)$$

The first-order condition for optimization is as follows:

(8) $$\frac{\partial \pi }{\partial E}=p_{c}g_{E}^{\prime }-h^{\prime }(E)=0$$

Solving this equation yields the optimal environmental governance investment:

(9) $$h^{\prime }(E^{*})=p_{c}g_{E}^{\prime }$$

Equation (9) implies that under a CSMA trading market, rising carbon prices (*p_c_*) or enhanced marginal contributions of *E* to CSMA compel coastal economic agents to increase environmental governance investments.

(3) Synergy Effect

When integrating economic and ecological considerations, coastal economic agents must determine optimal production factor inputs and environmental governance investment levels. Their objective is to maximize total profit and is expressed as follows:

(10) $$\max_{L,K,E}\;\pi =p_{q}f(L,K,E)+p_{c}g(f(L,K,E),E)-wL-rK-h(E)$$

Assuming short-term analysis with fixed capital *K*, the maximization problem requires satisfying the following two first-order conditions: production-side equilibrium and environmental-side equilibrium.

On the production side, the goal is to determine the optimal labor input, that is:

(11) $$\frac{\partial \pi }{\partial L}=p_{q}f_{L}^{\prime }+p_{c}g_{Q}^{\prime }f_{L}^{\prime }-w=0$$

In Equation (11), $p_{q}f_{L}^{\prime }$ denotes the marginal value product of labor, $p_{c}g_{Q}^{\prime }f_{L}^{\prime }$ captures the additional CSMA revenue from labor-induced output growth, and −w represents the marginal labor cost (i.e., wage rate). The production-side equilibrium condition is simplified as follows:

(12) $$\left(p_{q}+p_{c}g_{Q}^{\prime }\right)f_{L}^{\prime }=w$$

On the environmental side, the optimal ecological quality $E^{*}$ satisfies the following expression:

(13) $$\frac{\partial \pi }{\partial E}=p_{q}f_{E}^{\prime }+p_{c}\left(g_{Q}^{\prime }f_{E}^{\prime }+g_{E}^{\prime }\right)-h^{\prime }(E)=0$$

In Equation (13), $p_{q}\cdot f_{E}^{\prime }$ reflects the marginal production benefit of ecological quality; $p_{c}\left(g_{Q}^{\prime }f_{E}^{\prime }+g_{E}^{\prime }\right)$ represents the dual CSMA benefits (indirectly via output *Q* and directly via efficiency gains); and $h^{\prime }(E)$ is the marginal governance cost. The environmental-side equilibrium condition is reduced into the following expression:

(14) $$p_{q}f_{E}^{\prime }+p_{c}\left(g_{Q}^{\prime }f_{E}^{\prime }+g_{E}^{\prime }\right)=h^{\prime }(E)$$

Solving Equations (12) and (14) simultaneously allows analysis of equilibrium solutions $\left(Q^{*},E^{*}\right)$ under CSMA trading markets and varying carbon prices. On the production side, a higher carbon price $p_{c}$ increases the effective marginal benefits $\left(p_{q}+p_{c}g_{Q}^{\prime }\right)$. Given the diminishing marginal productivity of labor, enterprises must increase $L^{*}$ to maintain equilibrium, thereby elevating aquaculture output $Q^{*}$. On the environmental side, rising $p_{c}$ amplifies the total marginal benefits of ecological quality $p_{q}f_{E}^{\prime }+p_{c}\left(g_{Q}^{\prime }f_{E}^{\prime }+g_{E}^{\prime }\right)$. Due to the rising marginal cost of governance, enterprises are compelled to increase investments in environmental protection $E^{*}$. This investment further enhances the production function efficiency Q=f(L,K,E), enabling higher $Q^{*}$ at fixed input levels and establishing a synergistic pathway for marine economic–ecological co-development.

#### 3.3.3. Practical Cases of CSMA Trading

(1) Lianjiang: China’s First CSMA Transaction

In January 2022, Lianjiang County, Fujian Province, completed China’s inaugural CSMA transaction, trading 15,000 tonnes of carbon sink for CNY 120,000 (CNY 8/tonne) through the Xiamen Property Rights Trading Center. Supported by technical verification from the Third Institute of Oceanography, this transaction involved carbon sequestration from shellfish (e.g., oysters) and algae (e.g., kelp) farming. The county’s annual CSMA potential exceeds 400,000 tonnes, with shellfish contributing 90% due to large-scale mechanized aquaculture platforms. Post-transaction innovations included integrating CSMA with judicial remediation (e.g., offenders offsetting ecological damage by purchasing 7000 tonnes of CSMA) and financial instruments (e.g., “blue carbon loans” collateralized by CSMA rights). By 2023, cumulative transactions surpassed 60,000 tonnes, generating over CNY 400,000 in revenue.

(2) Ningbo: China’s First CSMA Auction

In February 2023, Ningbo’s Xiangshan County pioneered a CSMA auction, selling 2340.1 tonnes of algae-based carbon sink from Huangbizao Township’s coastal farms. Starting at CNY 30/tonne, the final price surged to CNY 106/tonne (total CNY 248,000), reflecting a 253% premium. This auction, facilitated by Ningbo Marine Institute, leveraged the region’s algal (23,400 tonnes/year) and shellfish (32,200 tonnes/year) CSMA potential. The high price underscored blue carbon’s scarcity and corporate demand for future carbon offsets, particularly amid expectations of China’s CCER market relaunch. Auction proceeds funded local marine habitat restoration, while participating firms (e.g., Zhejiang Yiduan Machinery) reserved credits for carbon-neutral product certifications.

(3) Zhangzhou: China’s First Municipal CSMA Repository

Zhangzhou established the nation’s first prefecture-level CSMA repository in December 2023, cataloging 240,000 tonnes of carbon sink from 17,000 hectares of shellfish (oysters and clams) and algae (kelp and Gracilaria) farms. Managed by Zhanglong Group, the repository employs blockchain-based traceability to prevent double-counting and issues “blue carbon tickets” for transactions. By February 2024, 1290 tonnes had been traded, including a cross-border sale of 878 tonnes of Indonesian Gracilaria carbon sink to Industrial Bank. Local governments integrated CSMA revenues into fiscal systems, allocating CNY 15,000 from Longhai District’s inaugural transaction to mangrove restoration. This centralized, data-driven model reduces verification costs, enhances market transparency, and enables scalable CSMA development—key prerequisites for integrating blue carbon into national emission trading systems.

These practical cases collectively validate the operational feasibility of the CSMA trading mechanism and its potential economic and ecological benefits. Lianjiang’s pioneering transaction exemplifies institutional innovation in bridging ecological governance with market incentives, while Ningbo’s auction model demonstrates the role of price discovery in reflecting blue carbon’s economic scarcity. Zhangzhou’s centralized repository further showcases how data-driven management enhances transparency and scalability. The alignment of economic returns (e.g., premium auction prices and diversified income streams) with ecological outcomes (e.g., habitat restoration funded by CSMA revenues) underscores the mechanism’s dual functionality. By integrating localized aquaculture practices with market frameworks, these cases highlight CSMA trading as a replicable pathway for transforming marine ecological services into tangible assets, fostering synergies between coastal economic development and environmental stewardship.

## 4. Discussion

### 4.1. China’s CSMA Exhibits Substantial Development Potential

The CSMA assessment spanning 2004–2023 reveals a distinct upward trajectory, with total CSMA more than doubling during this period. This trend reflects not only the continuous expansion of marine aquaculture but also highlights CSMA’s dual potential in ecological restoration and economic value realization. With the adoption of scientific farming practices and technological advancements, the carbon sink performance of marine aquaculture has improved incrementally, while the market value of CSMA has transitioned from an exploratory phase to a mature trading mechanism. A growing body of international research corroborates the role of coastal “blue carbon” resources in mitigating greenhouse gas emissions and enhancing marine ecosystem health [40,41]. Our findings reinforce the perspective that China possesses substantial untapped CSMA potential and outline a novel pathway for its low-carbon economic transition.

Compared to traditional blue-carbon systems, CSMA represents an emerging sequestration modality with distinct and increasingly evident advantages. Scholars emphasize that natural marine carbon sinks are limited by environmental and managerial constraints, whereas marine aquaculture—through large-scale standardized production and modern monitoring—achieves higher biomass densities and significantly enhances carbon sink rates [42,43]. Our analysis highlights pronounced species-level advantages and regional specialization patterns, indicating that optimizing species composition and adapting resource integration to local conditions can further enhance the aggregate sequestration efficiency of CSMA. Furthermore, well-functioning carbon markets enable the conversion of CSMA credits into economic incentives, which motivates producers to augment investments in technological innovation and ecological stewardship. This mechanism aligns with prior findings positioning CSMA trading as central to China’s strategy for alleviating escalating carbon-emission pressures [44].

A multi-perspective comparison of our data with existing literature underscores CSMA’s development potential across three primary dimensions. First, scale economies are evident: supportive policies and continuous technological innovation drive the sustained growth of total CSMA, providing a foundation for coastal restoration initiatives and a robust blue carbon market. Second, the realization of economic value through CSMA trading directly incentivizes technological upgrades and enhances environmental governance practices within marine aquaculture enterprises. Third, heterogeneous regional development paces have fostered diverse production models, creating opportunities for place-based management strategies and tailored policy design.

While marine aquaculture contributes positively to carbon sequestration, it is important to recognize that it also generates greenhouse gas (GHG) emissions and other carbon outputs throughout the production chain. The largest source of emissions stems from feed production—particularly when reliance on fish meal and fish oil involves wild-capture fisheries, intensive processing, and long-distance transport—leading to elevated CO_2_ emissions upstream of the farm [45,46]. During cultivation, uneaten feed and excreta accumulate in the water column and sediments, where microbial decomposition produces nitrous oxide (N_2_O) and methane (CH_4_), both with global-warming potentials far exceeding CO_2_ [47,48]. Moreover, the transformation of coastal habitats—such as mangrove or seagrass conversion—to establish pond systems releases previously stored “blue carbon”, further aggravating net carbon losses [49]. Energy consumption for water pumping, aeration, and processing equipment, often powered by fossil fuels, adds another layer of CO_2_ emissions, especially in high-intensity operations [47]. These challenges underscore the necessity of advancing sustainable farming practices and technological innovations to minimize emissions and ensure that marine aquaculture delivers net climate benefits.

### 4.2. Significant Species-Level and Regional Differences in CSMA Capacity

Our analysis reveals pronounced heterogeneity in CSMA capacity among cultured species, driven by variations in biological traits, farming practices, and production efficiencies. For instance, crabs exhibit the highest carbon sink coefficient (1.26) but account for merely 11.3% of total CSMA. Conversely, shellfish, with the lowest coefficient (0.088), dominate total CSMA (64.3%) owing to extensive large-scale cultivation. This asymmetry aligns with prior studies on interspecies functional differentiation in blue carbon systems [50,51]. Shrimp occupy an intermediate role, balancing carbon sink efficiency and production volume, which reflects a functional division of labor within marine aquaculture’s sequestration portfolio. As Yu et al. demonstrated [52], species-specific growth rates, metabolic traits, and farming technologies enhance the adaptability of CSMA capacities, providing a theoretical foundation for optimizing species composition and maximizing blue carbon benefits.

Regional disparities in CSMA capacity stem from variations in natural endowments, technological adoption, market orientation, and policy support among China’s coastal provinces. Our data reveal a pronounced tiered structure: Guangdong, Shandong, and Fujian dominate total and species-specific CSMA, reflecting robust economies, abundant marine resources, and advanced farming systems. In contrast, Hebei, Jiangsu, and Hainan lag significantly due to less favorable environmental conditions and resource constraints. This finding aligns with Guo et al. [53], who demonstrated that improvements in farming efficiency predominantly drive CSMA variations, whereas structural adjustments exert limited impact under stable aquaculture yields. Thus, provincial CSMA fluctuations reflect both inherent environmental disparities and the effectiveness of technological and managerial innovations. Regional policies should prioritize raising technical standards and optimizing farm structures to enhance CSMA capacities in underperforming regions.

Furthermore, species-level and regional disparities are interdependent and exhibit dynamic interactions. In shellfish-dominant zones (e.g., Shandong, Fujian, Liaoning), concentrated shellfish farming achieves exceptional sequestration outcomes. In shrimp-dominant regions (e.g., Guangxi and Hainan), high-density monoculture systems exhibit strong local efficiency but face seasonal and ecological instability. In dual-core regions such as Guangdong, advanced deep-water net-pen techniques and integrated aquaculture clusters enhance both production volume and overall CSMA through interspecies synergies. Existing studies emphasize that biodiversity and the synergistic integration of diversified farming systems are critical for maximizing CSMA outcomes [54,55]. To fully leverage CSMA’s potential in greenhouse gas mitigation, future research should prioritize cross-regional and cross-species coordination studies, incorporating quantitative assessments of species growth traits, farming practices, and environmental carrying capacities. China can achieve comprehensive enhancement of CSMA capacities—and thereby underpin sustainable low-carbon development and marine conservation strategies—only through optimized species configurations and the exploitation of comparative regional advantages.

### 4.3. CSMA Trading as the Principal Pathway for Realizing CSMA Value

As China progresses toward its low-carbon goals, CSMA—a core component of blue carbon resources—provides both a robust ecological solution and significant economic benefits. The inclusion of CSMA within carbon trading scopes, rather than other marine carbon sinks such as dissolved organic carbon (DOC) or particulate organic carbon (POC) storage, is grounded in two main considerations. First, from the perspective of property rights clarity, market-based trading requires unambiguous ownership of the traded asset. Owing to the fluid nature of seawater, establishing clear, enforceable rights over naturally occurring marine carbon sinks has proven difficult [56]. In contrast, the products derived from marine aquaculture have well-defined ownership—namely, the aquaculture operator—thus satisfying the prerequisite of clearly attributable property rights. Second, not all marine carbon sinks are eligible for trading under current carbon market rules, which stipulate that only net increments generated by projects following approved methodologies—and therefore exhibiting “additionality”—can be transacted. Endogenous marine biota, which function as part of the natural biological carbon pump, cannot be traded because they do not meet the additionality criterion. By contrast, carbon sequestration resulting from aquaculture activities represents a verifiable incremental increase in marine carbon sinks as regulated under approved methodologies, making CSMA uniquely qualified for inclusion in carbon trading schemes.

While natural marine carbon sinks face inherent scalability constraints, marine aquaculture achieves rapid biomass accumulation and enhanced carbon storage through intensification and modernization. The establishment of CSMA trading is thus pivotal for converting these ecological gains into economic value. A well-designed trading mechanism converts aquaculture-generated carbon sinks into tradable credits, allowing governments, enterprises, and stakeholders to share economic benefits via transparent market transactions. Studies demonstrate that mature carbon markets optimize resource allocation and incentivize firms to enhance environmental management and technological innovation [57,58,59]. This market incentive complements—and occasionally surpasses—traditional ecological compensation schemes, providing aquaculture operators with tangible economic incentives to engage in the low-carbon transition. Both theoretical and empirical evidence converge on the perspective that integrating CSMA into certified voluntary or compliance emission-reduction frameworks can effectively translate ecological outcomes into economic value, thereby establishing a mutually beneficial relationship between marine economic development and ecosystem protection across regional and national scales.

Furthermore, CSMA trading bridges the gap between marine economic activities and environmental stewardship, serving as a critical mechanism for achieving carbon neutrality. By transitioning from command-and-control to market-based mechanisms, carbon trading mobilizes enterprises and social stakeholders [60,61], fostering more efficient utilization of marine resources. As highlighted by Zhao et al. and Huang et al. [62,63], market-driven carbon trading improves the liquidity of carbon sink assets and accelerates the development of green finance and low-carbon technologies. However, realizing these benefits requires adapting CSMA trading to local contexts through differentiated policies that balance ecological safety with the exploitation of region-specific sequestration advantages. Future advancements in technology and governance frameworks are expected to transform CSMA trading into a comprehensive blue carbon marketplace, providing a sustainable model for marine ecosystem restoration and low-carbon economic development in China and beyond.

## 5. Conclusions

This study presented a comprehensive assessment of China’s marine aquaculture carbon sink (CSMA) over 2004–2023, identifying three distinct growth phases—initial fluctuation (2004–2008), rapid expansion (2008–2015), and optimization (2015–2023)—and quantifying a cumulative sequestration of 46.36 Mt. By species, shellfish dominated CSMA at 45%, followed by shrimp (24%), fish (15%), crab (11%), and algae (5%). Regionally, Guangdong, Shandong, and Fujian consistently led in total CSMA; Liaoning, Zhejiang, and Guangxi formed a second tier; and Hebei, Jiangsu, and Hainan remained at the lower end. Furthermore, each province displayed distinct species-dominance profiles: shellfish-dominant in Shandong, Fujian, and Liaoning; shrimp-dominant in Hebei and Hainan; shellfish-and-shrimp dual-cores in Guangdong and Guangxi; and a multifaceted profile in Jiangsu and Zhejiang.

To promote CSMA value realization, we designed a trading mechanism comprising three stages: identification of participants and monitoring evaluation in pre-transaction; platform development and price negotiation during transactions; and supervision and feedback adjustment in post-transaction. Based on the derivations from the effect analysis model and the illustrative case studies, we explore the potential economic and ecological benefits of CSMA trading.

To maximize the potential of CSMA trading, targeted policy interventions are essential. First, standardize quantification protocols by establishing unified methodologies for carbon sink measurement and verification, ensuring comparability across regions. This includes adopting IoT-based monitoring and blockchain traceability to reduce data disputes. Second, expand market participation by incentivizing private-sector engagement—for instance, allowing small-scale farmers to aggregate CSMA credits through cooperatives and enabling enterprises to use CSMA offsets in ESG reporting. Third, integrate CSMA into national carbon markets by aligning certification standards with China’s CCER framework, thereby attracting compliance-driven buyers from energy-intensive industries. Fourth, strengthen legal frameworks to clarify ownership rights over CSMA resources, particularly in overlapping jurisdictional zones, and enforce penalties for fraudulent trading. Finally, promote financial innovation through blue carbon bonds, insurance products, and cross-border trading pilots to enhance liquidity and global relevance.

Indeed, leveraging abundant CSMA resources and the national “dual-carbon” strategy, China has completed multiple pilot CSMA trades in Fujian and Zhejiang since 2022. These pilots confirm the practical feasibility of CSMA trading. However, most existing transactions have relied on government-led auctions or procurement tenders. Future efforts should harness market mechanisms to facilitate CSMA trades among enterprises, individual farmers, and other micro-level actors. In addition, conducting quantitative studies on the impacts of CSMA trading mechanisms, supported by enhanced data availability and comprehensive carbon balance accounting that considers the entire breeding process, should be prioritized in future research.

## Figures and Tables

**Table 1:** Accounting coefficient of carbon sink capacity of shellfish (unit: %).

Species	*R_d_*	*M_rt_*	*M_s_*	*cM_rt_*	*cM_s_*
Clam	52.55	1.98	98.02	44.90	11.52
Scallop	63.89	14.35	85.65	42.84	11.40
Oyster	65.10	6.14	93.86	45.98	12.68
Mussel	75.28	8.47	91.53	44.40	11.76
Ark Shell	37.99	9.63	90.37	45.86	11.29
Razor Clam	70.48	3.26	96.74	42.24	13.24
Other Shellfish	64.21	11.41	88.59	43.87	11.44

Note: All accounting coefficients are derived from the marine industry standard (HY/T 0305-2021), issued by the Ministry of Natural Resources of China on 9 February 2021.

**Table 2:** Accounting coefficient of carbon sink capacity of algae (unit: %).

Species	*Rs*	*W_ci_*
Kelp	20	31.20
Sea Lettuce	20	27.10
Gracilaria tikvahiae	20	28.40
Nori	20	41.96
Gracilaria lemaneiformis	20	31.93
Wakame	20	28.81
Agarophyte	20	26.37
Sargassum	20	30.97
Other Algae	20	30.36

Note: All accounting coefficients are derived from the marine industry standard (HY/T 0305-2021), issued by the Ministry of Natural Resources of China on 9 February 2021.

## Data Availability

Data are contained within the article.
